# Review of Journal of Cardiovascular Magnetic Resonance 2011

**DOI:** 10.1186/1532-429X-14-78

**Published:** 2012-11-18

**Authors:** Dudley J Pennell, John Paul Carpenter, David N Firmin, Philip J Kilner, Raad H Mohiaddin, Sanjay K Prasad

**Affiliations:** 1 CMR Unit Royal Brompton Hospital, Sydney Street, London, SW3 6NP, UK; 2 National Heart and Lung Institute, Imperial College, Exhibition Road, London, SW7 2AZ, UK

## Abstract

There were 83 articles published in the *Journal of Cardiovascular Magnetic Resonance* (JCMR) in 2011, which is an 11% increase in the number of articles since 2010. The quality of the submissions continues to increase. The editors had been delighted with the 2010 JCMR Impact Factor of 4.33, although this fell modestly to 3.72 for 2011. The impact factor undergoes natural variation according to citation rates of papers in the 2 years following publication, and is significantly influenced by highly cited papers such as official reports. However, we remain very pleased with the progress of the journal's impact over the last 5 years. Our acceptance rate is approximately 25%, and has been falling as the number of articles being submitted has been increasing. In accordance with Open-Access publishing, the JCMR articles go on-line as they are accepted with no collating of the articles into sections or special thematic issues. For this reason, the Editors feel it is useful to summarize the papers for the readership into broad areas of interest or theme, which we feel would be useful, so that areas of interest from the previous year can be reviewed in a single article in relation to each other and other recent JCMR articles [1]. The papers are presented in broad themes and set in context with related literature and previously published JCMR papers to guide continuity of thought in the journal. We hope that you find the open-access system increases wider reading and citation of your papers, and that you will continue to send your quality manuscripts to JCMR for publication.

## Cardiac volumes, function and mass

CMR is reasonably mature for assessment of cardiac function, with categorization by age decile, gender and body surface area for normal values for the left ventricle (LV) [2], right ventricle (RV) [3] and left atrium [4]. Although values for special groups are still being defined [5,6], a number of research papers are still being published for assessment of less common parameters of cardiac performance [7-9] as well as analysis software [10,11].

### Effect of lifestyle intervention plus rosiglitazone or placebo therapy on left ventricular mass assessed with cardiovascular magnetic resonance in the metabolic syndrome

Patients with the metabolic syndrome are often found to have left ventricular hypertrophy and raised LV mass [12]. Thiazolidinediones (rosiglitazone, pioglitazone and troglitazone) represent a group of insulin sensitizing agents which lower blood glucose levels by enhancing hepatic and peripheral glucose uptake as well as increasing free fatty acid uptake and storage in adipose tissue (thereby decreasing free fatty acid uptake in other tissues) [13]. Whether thiazolidinediones have a beneficial or adverse effect on LV mass has previously proved controversial. Roes reported the cardiovascular findings in a randomly chosen subset of patients involved in a much larger double-blind randomised controlled trial [14]. This was a trial looking at lifestyle intervention in combination with either rosiglitazone or placebo and its effect on carotid artery atherosclerosis in the metabolic syndrome. Lifestyle intervention resulted in a reduction of indexed LV mass in the placebo group, indicating reverse remodelling but there was no reduction in the rosiglitazone group and the authors suggest that rosiglitazone therapy may have inhibited this positive reverse remodelling.

### Strong cardiovascular prognostic implication of quantitative left atrial contractile function assessed by cardiac magnetic resonance imaging in patients with chronic hypertension

Increased left atrial size is a marker of left ventricular diastolic dysfunction and is a direct result of raised left ventricular end-diastolic pressure. It is a strong prognostic predictor of outcomes in patients with diastolic dysfunction but less is known about the prognostic significance of atrial contractile function. Over a median follow-up period of 19 months, Kaminski studied 210 patients with chronic hypertension who underwent a clinically indicated CMR scan for assessment of left ventricular function, myocardial ischemia or viability [15]. Left atrial contractile function was calculated from indexed biplane area-length measurements. The active component of atrial contractile function showed strong independent associations with major adverse cardiac events (MACE) (p < 0.0004), all-cause mortality (p < 0.0004), and non-fatal events (p < 0.0004) even after adjusting for age, gender, left atrial volume, and LV ejection fraction. This study adds strength to the growing literature on the prognostic value of CMR-derived cardiovascular parameters.

### Comparison of long and short axis quantification of left ventricular volume parameters by cardiovascular magnetic resonance, an ex-vivo validation

It is without doubt that one of the major advantages of CMR over other imaging techniques is the robust measurement of myocardial volumes and mass. High interstudy and interobserver reproducibility makes the technique ideally suited for longitudinal follow-up and can dramatically reduce sample size required for clinical trials [16]. Childs reported a comparison of short-axis derived ventricular volumes versus a novel technique using six radial long-axis steady state free precession (SSFP) cine sequences in explanted canine hearts and in 46 patients referred for CMR assessment [17]. Both short axis and long axis techniques were highly accurate but the rotational long axis approach proved more reproducible and time-efficient (with a 27% shorter evaluation time for experienced operators). A rotational long axis approach may therefore be a viable alternative for the clinical assessment of cardiac volumes, function and mass but would require a redesign of most of the current commercially available CMR analysis packages.

### Regional myocardial function after intracoronary bone marrow cell injection in reperfused anterior wall infarction – a cardiovascular magnetic resonance tagging study

This paper was a report of the CMR tagging substudy of the Autologous Stem Cell Transplantation in Acute Myocardial Infarction trial (ASTAMI), exploring the regenerative effects of intracoronary delivery of autologous mononuclear bone marrow stem cells [18]. Grid tagging using a FLASH gradient echo sequence was performed at 2-3 weeks and at 6 months after successful revascularisation with primary percutaneous intervention in 15 patients and 13 controls presenting with anterior ST-segment elevation myocardial infarction. The authors found an improvement in global and infarct zone strain in the control group which was greater than that observed in those who had received intracoronary stem cells. Conversely, LV mass decreased more in the control group than the stem cell group. Although the finding that this type of stem cell therapy does not strengthen regional or global myocardial function might seem somewhat disappointing at first glance, investigators in this field are focused on working out the correct stem cells to give and the optimal route of delivery. CMR will be at the forefront of that discovery.

### The relative atrial volume ratio and late gadolinium enhancement provide additive information to differentiate constrictive pericarditis from restrictive cardiomyopathy

The differentiation of restrictive cardiomyopathy from pericardial constriction can be challenging as both conditions may have similar imaging features but require a very different management strategy. Patients with surgically documented pericardial constriction (n-23), restrictive cardiomyopathy (n = 22) and normal subjects (n = 25) were included in this study [19]. Cheng et al. compared left and right atrial volumes using a relative atrial volume ratio (RAR) defined as the LA volume divided by the RA volume. Using a cut-off value of 1.32 for the RAR, the sensitivity for the detection of constriction was 82.6%, with a specificity of 86.4%. Septal bounce was identified in 95.7% of constriction patients but in none of the patients with restriction or the normal subjects. Late gadolinium enhancement was present in 31.8% of those with restrictive cardiomyopathy but absent in all constriction patients and normals. Non-invasive techniques such as CMR often rely on indirect findings and the potential development of robust diagnostic criteria with the ability to distinguish constriction from restriction is very welcome for the practicing clinician.

### Cardiovascular magnetic resonance myocardial feature tracking detects quantitative wall motion during dobutamine stress

CMR myocardial feature tracking is a new technique which attempts to track myocardial motion at voxel level from standard steady-state-free-precession cine acquisitions. The technique generates values for circumferential and radial strain, and is not currently validated or in clinical use. Schuster et al performed feature tracking on 10 normals at 1.5 T using the 4 chamber view [20]. Studies were performed at rest and during dobutamine stress. Strain was measurable in all patients and at rest and stress. Values increased significantly with dobutamine in parallel with the increase in ejection fraction. Observer variability was best for LV circumferential strain. The authors conclude that feature tracking may develop into a useful clinical tool with further development and validation.

### Right ventricular dysfunction is a predictor of non-response and clinical outcome following cardiac resynchronization therapy

Cardiac resynchronization therapy (CRT) is of proven benefit in advanced heart failure, but an important subset do not respond. CMR has been increasingly used to study patients requiring CRT [21]. Alpendurada et al. studied the incremental significance of right ventricular function in predicting benefit from CRT in 60 patients [22]. In a multivariate model, only RV ejection fraction and myocardial scar burden were significant predictors of response to CRT. Patients with RV EF < 30% had a particularly poor response rate (18.2%). The authors suggest that RV function should be measured in patients being evaluated for CRT.

## Flow evaluation and valve disease

The capability of CMR to measure cardiovascular flow contributes greatly to the versatility of CMR in clinical practice, and development of baseline flow corrections [23], and automated analysis software [24] continues. This has greatest application in valve disease [25-27] but it is also often used in disease of the aorta [28,29], lungs, coronaries and in congenital heart disease.

### Cardiovascular magnetic resonance for the assessment of patients undergoing transcatheter aortic valve implantation: a pilot study

Correlation and agreement between transthoracic echocardiography (TTE) and CMR in the assessment of aortic root dimension and LV morphology and function were studied in 49 patients undergoing transcatheter aortic valve implantation (TAVI) [30]. There was a good correlation between TTE and CMR in terms of annulus size (R^2^ = 0.48, p < 0.001), left ventricular outflow tract (LVOT) diameter (R^2^ = 0.62, p < 0.001) and left ventricular ejection fraction (LVEF) (R^2^ = 0.47, p < 0.001) and a moderate correlation in terms of aortic valve area (AVA) (R^2^ =0.24, p < 0.001). CMR generally tended to report larger values than TTE for all measurements. Bland-Altman analysis indicated that the 95% limits of agreement between TTE and CMR ranged from -5.6 mm to + 1.0 mm for annulus size, from -0.45 mm to + 0.25 mm for LVOT, from -0.45 mm2 to + 0.25 mm2 for AVA and from -29.2% to 13.2% for LVEF. CMR represents a viable complementary imaging method to TTE in elderly patients being considered for TAVI.

### Pseudoaneurysm of the left ventricle following apical approach TAVI

This is an interesting case report of a pseudoaneurysm of the left ventricle following trans-apical TAVI approach in an elderly lady with severe aortic stenosis [31]. A 23 mm Edwards Scientific Sapien XT valve prosthesis (a bovine tissue valve inserted on a cobalt chromium frame) was implanted with immediate transoephageal echocardiography and fluoroscopic imaging demonstrated excellent seating of the valve, subsequently confirmed angiographically. Two days later the patient developed isolated electrocardiographic evidence of pericarditis with minimal associated chest pain. Transthoracic echocardiography demonstrated a well seated aortic prosthesis and a 0.8 cm pericardial effusion with no tamponade. The effusion was not drained and her electrocardiographic changes settled over the next few days. Three months later, transthoracic echocardiography showed a discrete pseudoaneurysm with late gadolinium myocardial enhancement. She was managed conservatively with regular surveillance.

### Contrast-enhanced CMR in patients after percutaneous closure of the left atrial appendage: A pilot study

There are currently a number of commercially available devices for left atrial appendage (LAA) closure to prevent the formation of thrombus and risk of stroke in atrial fibrillation. However, imperfect device placement or suboptimal ‘fit’ causing a residual ‘leak’ into the LAA has the potential to allow thrombus to form. This would mandate that the patient remains on long-term anticoagulation medication, thereby negating the potential benefit from device implantation. Mohrs’ pilot study of 7 patients with three different device occluders (Watchman, PLAATO and ACP) assessed the feasibility of using CMR to detect residual leaks around the devices [32]. First-pass perfusion using SSFP was followed by a 3D Turbo-FLASH sequence. The authors found a very high percentage with residual leaks (57%) but noted that the study only included patients in whom problems of device malposition or residual leaks were suspected. There are still questions to be answered (including whether CMR is able to detect small thrombi on the device surface) but this very interesting approach is likely to inform future device design and procedural quality control.

### Sequence optimization to reduce velocity offsets in cardiovascular magnetic resonance volume flow quantification - A multi-vendor study

This manuscript describes a follow-up study to the multi-vendor, multi-centre study by Gatehouse in which they measured offsets in phase contrast velocity mapping studies [33]. In this paper, the effects on velocity offsets of changes to the exam protocol parameters were examined, with the goal of finding a method to minimise offsets at the acquisition stage without the need of post-processing correction schemes [34]. Although important, the conclusion was perhaps overly negative in stating that with current systems there was no generic protocol which resulted in acceptable flow offset values.

## Congenital and pediatric heart disease

Congenital heart disease remains a major indication for CMR, and makes particular contribution in the assessment of the major vessels [35], shunt assessment [36], in complex anatomy, and increasingly in children and neonates [37]. A wide range of clinical scenarios in congenital heart disease were covered in JCMR in 2011.

### Cardiovascular magnetic resonance tagging of the right ventricular free wall for the assessment of long axis myocardial function in congenital heart disease

Quantification of right ventricular myocardial function and any changes of it over time are important issues in congenital heart disease. Volumetric calculation of right ventricular (RV) ejection fraction is time consuming to acquire and analyse, and inter-study reproducibility is suboptimal. In this study, a linear tag orientated perpendicular to the basal free wall of the RV was used to measure long axis systolic displacement [38]. The method was quick to acquire and analyse, reproducible and showed more significant differences between the clinical groups studied than did RV ejection fraction. This suggests it has potential as an additional method, particularly for longitudinal comparisons.

### 3D Echo systematically underestimates right ventricular volumes compared to cardiovascular magnetic resonance in adult congenital heart disease patients with moderate or severe RV dilatation

Three dimensional echo is a relatively new technique which promises to offer a rapid alternative to CMR for the examination of the right heart. In this study, comparison of 3D echo with CMR measurements of RV volumes and function was undertaken in patients with varying degrees of RV dysfunction resulting from congenital heart disease [39]. The echo technique was found to underestimate RV volumes significantly, especially in patients with more severe dilatation and dysfunction. This lead the authors to conclude that 3D echo, as implemented, was not ready for routine clinical use for the assessment of more than mild dilatation of the RV.

### Assessment of pulmonary veins after atrio-pericardial anastomosis by cardiovascular magnetic resonance

Surgical anastomoses of pulmonary veins are prone to re-stenosis, especially if attempted in the small, delicate veins of infants. For this reason, atrio-pericardial anastomosis uses a pericardial pouch to create a large communication between the left atrium and the pulmonary veins. It avoids direct suturing of the pulmonary veins during the repair of various congenital malformations. This study reviewed CMR assessments of this approach, performed in 31 of the 103 patients undergoing the procedure [40]. Findings were compared with those by echocardiography, concluding that CMR gave a more thorough assessment. Even after the atrio-pericardial anastomotic technique, stenosis of the pulmonary veins remained a common complication, being identified in 12 of the 31 CMR studies performed.

### The role of cardiovascular magnetic resonance in candidates for fontan operation: proposal of a new algorithm

Fontan surgery, performed for children with only one effective ventricle, involves reconnection of the systemic venous returns to the pulmonary arteries so that the one effective pump delivers flow to the systemic and then the pulmonary vasculature in series. The procedure requires careful pre-operative planning. After reviewing the investigations performed and their findings in 44 patients undergoing Fontan surgery, the authors of this paper propose a pre-procedure investigative-diagnostic algorithm [41]. They found that a combination of trans-thoracic echocardiographic and CMR investigations can be used to triage patients, some of whom can then proceed to surgery without the need for cardiac catheterisation.

### The role of cardiovascular magnetic resonance in pediatric congenital heart disease

CMR is rapidly coming to be regarded as an indispensible imaging and investigative modality in paediatric cardiology. However, imaging small children with congenital heart disease is challenging. This paper provides an authoritative and clearly written review of the technical adjustments, imaging protocols and applications of CMR in the paediatric population [42].

### Delayed onset of tricuspid valve flow in repaired tetralogy of Fallot: an additional mechanism of diastolic dysfunction and interventricular dyssynchrony

Those who routinely image patients with dilated and/or pressure loaded RVs are likely to have noticed signs of prolonged systole of the RV relative to that of the LV. This study used phase contrast velocity mapping to record the time course of tricuspid and mitral flow in 31 children with repaired tetralogy of Fallot [43]. The authors found delayed onset of tricuspid flow to be common in this group and associated with reduced RV ejection fraction. This manifestation of interventricular dyssynchrony may be one of several possible mechanisms of ventricular diastolic dysfunction.

### Quantitative cardiovascular magnetic resonance in pregnant women: cross-sectional analysis of physiological parameters throughout pregnancy and the impact of the supine position

CMR study of an expectant mother’s haemodynamics is feasible and acceptable during mid to late pregnancy. It may be more comfortable in a left lateral than the supine position. This study of healthy pregnant women, 6 in mid pregnancy and 8 late in pregnancy, compared haemodynamic parameters and the effects of position on them [44]. It concluded that, from as early as 20 weeks, the left lateral position appears to be preferable. This was based on the greater stroke volumes and cardiac outputs measured in this position than supine, which presumably tends to put pressure on systemic veins from the lower body.

### Comparison between cardiovascular magnetic resonance and transthoracic Doppler echocardiography for the estimation of effective orifice area in aortic stenosis

This study compared CMR and echocardiographic approaches to assessments of aortic stenosis (AS) using the continuity approach that incorporates calculations of volume flow at left ventricular outflow tract (LVOT) level, and the post stenotic jet velocity time integral [45]. CMR showed the LVOT cross-section to be ovoid, not circular, and that Echo, which typically recorded the smaller ovoid diameter, tended to underestimate this area. On the contrary, CMR tended to underestimate jet velocities relative to echo. These presumed errors tended to cancel out, resulting in little mean difference between the echo and CMR calculations of effective orifice area. However, CMR was associated with less intra- and inter- observer variability of measurement. Some readers may prefer to measure stenotic orifice area directly by planimetry from one of a stack of appropriately acquired CMR cines, aligned to transect the jet near its origin. This relatively straightforward approach was not investigated in the study reported.

### Pulmonary flow profile and distensibility following acute pulmonary embolism

This study used phase contrast CMR to record pulmonary artery flow in groups of patients with and without pulmonary embolus detected by computed tomography (CT) about 6 months previously [46]. Both groups had been investigated for suspicion of PE. The sample sizes were relatively small and the CMR measures followed the initial months of therapy. Although RV functional differences had been documented by initial CT, no significant differences were found in the amounts or the temporal profiles of pulmonary flow by the CMR studies. It remains unclear whether the lack of difference depended on the time interval and the therapy given. Only one of the patients had evidence of pulmonary hypertension at the time of CMR, which recorded the characteristic notch or depression in mid systolic flow and a reduction of peak velocity that are expected with elevated pulmonary vascular resistance.

### Repaired tetralogy of Fallot: the roles of cardiovascular magnetic resonance in evaluating pathophysiology and for pulmonary valve replacement decision support

Tetralogy of Fallot is one of the commonest and most important congenital heart conditions whose management after surgical repair calls for serial follow up by CMR. The author gives a comprehensive and thoughtful review of the methods and interpretation of CMR studies in relation to the pathophysiology of condition [47]. This review is recommended for reference and for explanations of methods and measurements that are currently considered to contribute most to clinical decision making.

### Comprehensive 4D velocity mapping of the heart and great vessels by cardiovascular magnetic resonance

Four dimensional (4D) phase contrast velocity mapping is a relatively novel and impressive CMR technique. It entails the acquisition of all 3 directional components of velocity for voxels distributed in all 3 dimensions of space, triggered over many heart cycles to represent multiple phases of the cardiac cycle. This paper reviews and illustrates the methods used for the acquisition, visualization, and quantification of 4D velocity datasets, which typically takes ten or more minutes [48]. Although more rapid and user-friendly strategies for acquisition and analysis may be needed before 4D velocity acquisitions come to be adopted in routine clinical CMR, their capacity to measure multidirectional flows throughout a study volume has contributed novel insights into cardiovascular fluid dynamics in health and disease.

### Cardiovascular magnetic resonance findings in repaired anomalous left coronary artery to pulmonary artery connection (ALCAPA)

Secinaro reported the use of CMR in a series of 6 patients at Great Ormond Street Hospital, London with clinical suspicion of ischemia following surgical repair for anomalous left coronary artery to pulmonary artery connection (ALCAPA) [49]. The mean age was 15.3 ± 4.2 years and half were female. Their protocol included volumes and function, late gadolinium, adenosine stress perfusion and 3D whole heart acquisition to assess coronary artery origins. In this cohort, basal anterolateral subendocardial myocardial fibrosis was a characteristic finding. Stress perfusion identifed reversible ischemia in 3 patients which was indicative of left coronary artery occlusion, confirmed at coronary angiography in all 3. This study confirms CMR as a reasonable non-invasive first-line investigation for the assessment of such patients.

## Iron overload cardiomyopathy

Since the first data on myocardial T2* CMR published in 2001 [50], there has been substantial and rapid progress in the use of this technique for patient benefit, with direct calibration to myocardial iron concentration [51], the association of myocardial T2* < 10 ms with adverse cardiac outcomes [52], and a reduction in cardiac mortality [53]. These 5 JCMR papers in 2011 continue to define the role of T2* in multiply transfused patients.

### On improvement in ejection fraction with iron chelation in thalassemia major and the risk of future heart failure

This paper analyzes the importance of increases in ejection fraction during treatment with iron chelators for myocardial siderosis on the future likelihood of developing heart failure, which is the most dangerous complication of multiple transfusions as the historical mortality is high once heart failure develops [54]. The premise behind the analysis is that both the absolute level and the trajectory of ejection fraction are useful markers of heart failure risk. A UK database was analyzed for patients with at least two CMR scans (with T2* and ejection fraction) with follow-up for development of heart failure. A total of 315 patients were included in the analysis, and patients were stratified into normal or reduced baseline ejection fraction. Statistical modelling showed that an improvement in ejection fraction was associated with a significantly lower risk of developing heart failure irrespective of baseline ejection fraction. However, the absolute risk of developing heart failure was greater in patients with lower ejection fraction. Using data on ejection fraction change from randomized controlled trials, the authors showed that the ejection fraction changes seen with oral deferiprone were associated with a 25.5% to 46.4% reduction in risk of developing heart failure. This is important because other iron chelators are not associated with such increases in ejection fraction despite improving myocardial T2*. The authors suggest that this difference between the iron chelators might be explained by the preferential relief by deferiprone of iron-related mitochondrial dysfunction.

### Value of black blood T2* cardiovascular magnetic resonance

The CMR sequence used to measure myocardial T2* has gone incremental improvement since the first use of a non-breathold sequence which was influenced by tissue T1 in 2001. A single breathold multi-echo sequence was introduced in 2003 and was shown to be reliable and reproducible [55]. However, these white blood sequences were subject to artefactual signal from the white blood pool smeared in the phase encode direction. This was problematic in hearts with heavy iron loading and at the longer echo times. He et al introduced the dark blood T2* sequence in 2007 [56] and in this paper Smith compares the white and black blood sequences [57]. The authors show that the black blood sequence had significantly improved intra-observer, inter-observer, and inter-study reproducibility. In addition, the black blood sequence has fewer imaging artefacts. Therefore, this sequence is recommended for clinical use.

### Low prevalence of fibrosis in thalassemia major assessed by late gadolinium enhancement cardiovascular magnetic resonance

Post-mortem reports in the 1960s and 1970s showed that patients dying of myocardial siderosis had severe replacement myocardial fibrosis. If this were the case today, it would be expected that heart failure related to myocardial siderosis would be irreversible. However, recent studies with aggressive intravenous iron chelation [58] have shown that it is possible to survive siderotic heart failure and regain normal cardiac function [59]. Kirk examined 45 thalassemia major patient using late gadolinium enhancement and found only one patient with enhancement [60]. No patient with a history of heart failure or low ejection fraction had LGE. The authors conclude that replacement myocardial fibrosis is unusual in the modern era, possibly due to the influence of increased use of transfusions, lower infection rates, or increased use of iron chelation treatments.

### Effect of deferiprone or deferoxamine on right ventricular function in thalassemia major patients with myocardial iron overload

Considerable attention has been paid to the effect of iron overload on the left ventricle, but little to its effects on the right ventricle. Recent work has shown normal right ventricular function in thalassemia [61], and its decline with myocardial iron loading [62]. This paper by Smith shows the effect of the iron chelators deferiprone and deferoxamine on right ventricular function [63]. Deferiprone increased right ventricular ejection fraction and reduced the end-systolic volume. Deferoxamine however, had no effect on right ventricular volumes or ejection fraction. The effects of the oral drug deferiprone with significantly superior to those of the injectable deferoxamine, and may contribute to the improved mortality seen with deferiprone.

### Iron overload in polytransfused patients without heart failure is associated with subclinical alterations of systolic left ventricular function using cardiovascular magnetic resonance tagging

Although myocardial T2* has been successfully introduced into clinical practice, it is not as available as it needs to be in lower income countries where thalassemia is prevalent. Seldrum examines CMR tagging as a possible alternative measure to detect myocardial iron [64]. In 10 patients with myocardial T2* < 10 ms, the authors showed that subclinical alterations in cardiac function of which left ventricular twist was the earliest to show abnormality and correlated best with T2*. Although this is of interest, in clinical practice, myocardial T2* is more specific for siderosis and tagging is less available and more complicated to analyse than myocardial T2*.

## Cardiomyopathy

CMR of patients with cardiomyopathy has become a leading referral over the last 5 years and there has been considerable interaction with cardiovascular geneticists as CMR offers high fidelity phenotyping [65], that augments genetic discovery [66]. CMR physicians have also started to work closely with electrophysiology colleagues to assess arrhythmic and sudden death risks [67]. Conditions related to cardiomyopathy [68,69], and rare forms of cardiomyopathy [70,71], are being studied and common themes of myocardial fibrosis development, pattern of deposition, strain patterns [72] and association with outcomes are being established [73]. CMR techniques continue to develop [74], most recently the technique of equilibrium T1 mapping after gadolinium to assess the interstitial compartment has become popular [75], although this is not straightforward and clinical application is not yet established. T2 mapping is also being investigated for its utility [76], and may be useful in combination with late gadolinium enhancement in conditions such as myocarditis [77].

### Hypertrophic cardiomyopathy and ultra-endurance running - two incompatible entities?

Hypertrophic cardiomyopathy (HCM) is often described as associated with a reduced exercise capacity, perhaps best demonstrated by a reduced peak VO2. It is also often considered that in patients with unexplained LV hypertrophy, a suboptimal VO2 is a hallmark of HCM rather than hypertensive disease. This case study reports an asymptomatic male athlete with 25 years of ultra-endurance competition, with genetically confirmed HCM phenotypically manifesting with LVH, a small LV cavity together with repolarisation abnormalities suggestive of HCM [78]. The authors speculate that despite documented asymmetric hypertrophy and focal myocardial fibrosis in the basal anteroseptal and inferoseptal walls, it is suspected that the athlete is able to run ultra-marathons due to a compliant LV with normal diastolic and systolic parameters, which is able to augment stroke volume.

### Increased left ventricular torsion in hypertrophic cardiomyopathy mutation carriers with normal wall thickness

In concert with major developments in genetic techniques an important group of patients are those that are gene positive and phenotypically negative, particularly in HCM. What is controversial is determining early markers of disease before the phenotype has manifested. In this study of 17 HCM mutation carriers, and normal wall thickness, using tissue tagging it was observed that there was increased LV torsion as well as endocardial circumferential strain and torsion-to-endocardial-circumferential-shortening ratio, which reflects the transmural distribution in contractile function [79]. Further work is required to determine if this may offer a potential therapeutic target.

### Myocardial extravascular extracellular volume fraction measurement by gadolinium cardiovascular magnetic resonance in humans: slow infusion versus bolus

Detection of interstitial fibrosis is one of the most exciting fields in CMR at present. There is still much controversy over sequence types, and in particular whether slow infusion carries significant advantage over the more practical bolus option. In this study of 10 volunteers, serial Ve measures were compared across the 2 methods [80]. Importantly, serial measures of Ve did not differ significantly between the constant infusion and bolus methods. There is strong correlation of readings to age. The findings of this work support development of T1 mapping for extracellular volume fraction (ECV) using the more pragmatic bolus approach that is also likely to simplify clinical application.

### Myocardial T1 and extracellular volume fraction mapping at 3 Tesla

Late gadolinium enhancement (LGE) has been the standard of reference for detecting focal myocardial fibrosis in clinical however, it relies on the differences in signal intensity between scarred and adjacent normal myocardium to generate image contrast. T1 mapping, is a promising quantitative method for detecting diffuse myocardial fibrosis. It has potential to provide significant insights into myocardial function. In this study, after validation of MOLLI sequences at 3 T in Phantoms, the authors present values for myocardial and blood T1 pre and post gadolinium contrast at 3 T [81]. At 3 T, post-gadolinium ECV is stable between 8.5 and 23.5 minutes after gadolinium injection.

### The clinical impact of late gadolinium enhancement in Takotsubo cardiomyopathy: serial analysis of cardiovascular magnetic resonance images

Takotsubo cardiomyapthy, also known as a stress-induced cardiomyopathy, refers to the transient LV apical ballooning observed after some form of stress that usually resolves. Part of the diagnosis assumes unobstructed coronaries. There is controversy over the presence of fibrosis. In this study, they observe that in 8 patients scanned serially in the sub-acute and late phase (1.5 and 6 months later), the presence of LGE in the subacute phase was associated with greater disease severity and more prolonged recovery [82]. These findings, if supported by larger studies would help stratify what is increasingly recognised as a heterogenous cohort of patients and presentations.

### Cardiovascular magnetic resonance in wet beriberi

Wet Beriberi is one of four clinical syndromes associated with Thiamine (Vitamin B1) Deficiency. Wet beriberi has varying degrees of cardiovascular involvement. In its most fulminant form, it is characterized by hypotension, tachycardia and lactic acidosis. If untreated, patients die within hours from circulatory collapse and pulmonary edema. This condition often goes unrecognized since it is easily confused with other illnesses. This is the first report demonstrating the CMR finding of myocardial edema associated with wet beriberi and uses a T2 mapping technique to gauge quantitative assessment [83]. Whilst an unusual case, it has relevance at a global level to those who encounter such patients and shows the application of T2 mapping in myocarditic settings.

### Cardiovascular magnetic resonance of cardiomyopathy in limb girdle muscular dystrophy 2B and 2I

Limb-girdle muscular dystrophy (LGMD) comprises a group of genetically-heterogeneous disorders that present with variable skeletal and cardiac muscle involvement. LGMD produces progressive weakness of proximal shoulder-girdle or pelvic muscles with a wide range of phenotypic expression, severity, and age of disease onset. What is less clear is the extent and degree of cardiac involvement. In this study, consecutive patients with genetically-proven LGMD types 2I (n = 7) and 2B (n = 9) and 8 control subjects were enrolled [84]. All subjects underwent CMR with cine imaging for left ventricular volume and ejection fraction measurement, vector velocity analysis of cine data to calculate myocardial strain, and late post-gadolinium enhancement imaging to assess for myocardial fibrosis. The majority of patients with LGMD of 2 subtypes - 2B, and 2I - in this cohort showed normal LV size, global systolic function and peak systolic circumferential strain. However, there was evidence of subclinical myocardial fibrosis in 57% of subjects with LGMD2I and 33% of subjects with LGMD2B. This abnormality was accompanied by diastolic dysfunction in a number of patients. Overall, the prevalence of advanced cardiomyopathy in patients with LGMD2I and LGMD2B appears to be limited in this cohort of patients, but subclinical fibrosis and diastolic dysfunction do occur and may warrant use of cardioprotective medical therapies.

### Late gadolinium enhanced cardiovascular magnetic resonance of lamin A/C gene mutation related dilated cardiomyopathy

The lamin A/C gene (LMNA) is so far the most significant disease gene identified clinically for dilated cardiomyopathy (DCM). It has been estimated that LMNA mutations cause up to 10% of familial DCM. The penetrance of the LMNA mutations causing cardiomyopathy is nearly complete. It is associated with an increased risk of sudden death and heart-failure. The main aim of this study was to characterise myocardial fibrosis, regional wall motion abnormalities, ventricular dilatation, longitudinal LV systolic function and global function with LGE CMR in asymptomatic or mildly symptomatic carriers of LMNA mutations causing DCM [85]. They also looked at the possible association between the localisation of myocardial fibrosis and the conduction abnormalities documented with electrocardiography. This is a relatively small study of 17 patients but what was interesting was that 88% had demonstrable myocardial fibrosis. Where present, there was an association with conduction abnormalities. A high proportion also had mild LV dilatation, impairment in function or longitudinal systolic function. As our genetic capabilities expand, the close link between genotype and phenotype is likely to yield interesting and important findings. It will also be interesting to see the phenotypic manifestations and subsequent clinical implications of the newly identified titin mutation that has been identified to account for about 30% of cases of familial DCM.

### Cardiac resynchronization therapy guided by late gadolinium-enhancement cardiovascular magnetic resonance

Cardiac resynchronization therapy has become an important mainstay in the management of patients with heart-failure. Unfortunately, about 30% of patients do not seem to show a clinical response. As a relatively expensive form of treatment, there is a need for better stratification of Patient selection and also to deploy better methods of optimizing lead placement at the time of implantation. In this study, the authors assessed whether the use of late gadolinium enhancement CMR scan to guide deployment of the LV lead in a non-scarred segment of the LV free wall leads to a better long-term outcome from CRT than using a conventional implantation approach [86]. This is a large study of 559 patients with heart-failure due to both an ischaemic related and non-ischaemic basis. Implantations were either guided (+CMR) or not guided (-CMR) by LGE-CMR prior to implantation. Fluoroscopy and LGE-CMR were used to localize the LV lead tip and myocardial scarring retrospectively. Clinical events were assessed in three groups: +CMR and pacing scar (+CMR + S); CMR and not pacing scar (+CMR-S), and; LV pacing not guided by CMR (-CMR). With longterm follow-up, over a maximum period of 9 years, the + CMR + S group had the highest risk of death from pump-failure and sudden death, compared to the other cohorts, and lowest in the + CMR-S group. These findings highlight the potential for CMR to guide CRT lead deployment but also that pacing scarred tissue is associated with a much worse outcome – presumably due to provoking re-entrant arrhythmias.

### Cardiovascular magnetic resonance in cardiac sarcoidosis with MR conditional pacemaker in situ

One of the main challenges confronting CMR is the burgeoning number of patients with conventional pacemakers/devices implanted. Whilst under certain restricted conditions, some sites are proceeding with CMR in these patients, and where there is an overwhelming benefit to risk balance, most sites remain conservative in their approach. For this reason, there has been great interest in MR-conditional devices. In this interesting case report, Quarta and colleagues describe how the management of a Patient with sarcoid and such a device, was facilitated by being able to perform CMR on the heart and secondly the impact of their findings [87]. Image quality was excellent and supports the case that there should be growing use of MR-conditional devices to ensure we do not preclude patients from deriving the benefits of CMR.

### Mild hypothermia delays the development of stone heart from untreated sustained ventricular fibrillation - a cardiovascular magnetic resonance study

Manoeuvres to improve outcomes following a ventricular fibrillation (VF) arrest are likely to improve the current suboptimal outcomes. In this study of 14 swine randomised to normothermia or hypothermia, the latter reduced the early LV dilatation typically seen and importantly, delayed the onset of stone heart thereby extending a known, morphologic limit of resuscitability [88]. These findings have important implications to algorithms for managing a VF arrest.

### T2-weighted cardiovascular magnetic resonance in acute cardiac disease

This excellent review assesses the impact of T2-weighted imaging in the assessment of acute cardiac disease [89]. Applications include the assessment of myocardial salvage as well as detection of acute myocarditis. Key strengths lie in its combination with late enhancement imaging. Whilst much focus has been on its diagnostic value, important newer areas are quantifying the area at risk and providing a quantitative means of assessing disease burden to determine therapeutic efficacy and gauge risk. Future work needs to focus on improving sensitivity, better quantification, timescale of changes, and what the true incremental value is for example in ACS in large well planned outcome studies.

### Effects of steroids and angiotensin converting enzyme inhibition on circumferential strain in boys with Duchenne muscular dystrophy: a cross-sectional and longitudinal study utilizing cardiovascular magnetic resonance

Whilst it is well recognised that Duchenne muscular dystrophy (DMD) carries an increased risk of cardiac morbidity, treatment of this cohort of patients suffers from a lack of systematic prospective clinical trials. In this study, the authors identified patients for inclusion in one of two treatment groups [90]. Group A was only treated with corticosteroids (either deflazacort or prednisone). Group B was being treated with corticosteroids plus ACEi (lisinopril or enalapril) or ARB (losartan). All patients in Group B had been treated with ACEI/ARB for a minimum of 12 months prior to CMR, and all patients in both groups had been treated with corticosteroids for at least 12 months prior to CMR. Initiation of corticosteroids and ACEI/ARB was determined exclusively by treating physician preference and were not based on CMR results. DMD boys not treated with corticosteroids or treated with beta blockers were excluded. Limitations include the lack of randomisation, physician preference in treatment, retrospective nature and lack of control. Notwithstanding, in 171 patients – a large cohort, there was no clear difference in slowing the decline in cardiac function. It highlights the need for ongoing work to look at more novel or targeted treatment strategies.

### Prevalence and distribution of regional scar in dysfunctional myocardial segments in Duchenne muscular dystrophy

Duchenne muscular dystrophy (DMD), is the most common of the muscular dystrophies. It has an incidence of 1 in 3,500 males. It is an X-linked recessive disorder resulting from a disabling mutation of the gene encoding dystrophin, a sarcolemmal protein found in skeletal and cardiac muscles. There is progressive skeletal muscle weakness with loss of ambulatory ability in the teenage years. Death is usually due to cardiac or respiratory failure, and distinctive pathologic findings have been noted. As noted by the authors, with improvements in overall management and respiratory treatment, there has been increasing focus on the prevention and treatment of cardiac disease in this condition. In this study, strain using myocardial tagging and fibrosis patterns were determined in a cohort of 16 patients with DMD [91]. There was also correlation with echo assessed segmental strain. Scar tissue was found to most common in the inferio, inferolateral and anterolateral walls, although seen in other regions as well. The relationship between scar and circumferential strain is however a little more complex with high negative but lower positive predictive for Ecc in the determining segments with scar.

### Presence of mechanical dyssynchrony in Duchenne muscular dystrophy

As noted above, DMD carries a high morbidity and mortality rate with cardiac dysfunction an important cause. In this large study of 236 Patients with DMD, dysynchrony was assessed based on timing of CMR derived circumferential strain (ecc) [92]. The calculated indices included cross-correlation delay (XCD), uniformity of strain (US), regional vector of variance (RVV), time to maximum strain (TTMS) and standard deviation (SD) of TTMS. Abnormal XCD value was defined as > normal + 2SD. As noted by the authors, there was overall low prevalence of circumferential dyssynchrony in the entire DMD population; it increased to 17.1% for patients with abnormal EF and to 31.2% in the most advanced stage (abnormal EF with fibrosis). All but one DMD patient with mechanical dyssynchrony exhibited normal QRS duration suggesting absence of electrical dyssynchrony. The calculated US and RVV values (0.91 ± 0.09, 1.34 ± 0.48) indicate disperse rather than clustered dyssynchrony. Patients also had a high prevalence of lateral wall fibrosis although usually in advanced disease. These findings suggest that CRT therapy is unlikely to offer much benefit in this population of patients.

## Atheroma and vascular

CMR is well suited to characterising the atherosclerotic arterial wall. Research has been focussed on early detection [93,94], pathogenesis [95-97], the monitoring of response to treatment, and relation to outcomes [98], rather than stenosis assessment for which other techniques are widely used. Vessel wall CMR, magnetic resonance angiography and other techniques benefit from the use of 3 T and higher fields [99,100]. The papers in this section illustrate the variety of ways that CMR can be used to investigate vascular disease.

### Age determination of vessel wall hematoma in spontaneous cervical artery dissection: A multi-sequence 3 T cardiovascular magnetic resonance study

This is an elegant prospective observational study in 35 patients with first time spontaneous cervical artery dissection to determine the age of vessel wall hematoma using multiple-weighted CMR on a 3 T scanner [101]. The manuscript is well written and data are well presented. The mean age of spontaneous cervical artery dissection (sCAD) was 2.0, 5.8, 15.7 and 58.7 days in patients with acute, early subacute, late subacute and chronic vessel wall hematoma (VWH) as classified by CMR (p < 0.001 for trend). Agreement was moderate between VWH types in this study and the previously proposed time scheme of signal evolution for cerebral hemorrhage, Cohen's kappa 0.43 (p < 0.001). There was a strong agreement of CMR VWH classification compared to the time scheme which was proposed for carotid intraplaque hematomas with Cohen's kappa of 0.74 (p < 0.001).

### Magnetization transfer magnetic resonance of human atherosclerotic plaques ex vivo detects areas of high protein density

This study investigates the use of magnetization transfer contrast (MTC) for atherosclerotic plaque characterization at 11.7 T [102]. High-resolution (0.047 × 0.047 × 0.5 mm) ex-vivo MTC MRI of human carotid artery specimens obtained from endarterctomy was performed. The main findings are that the magnetization transfer contrast ration (MTR = [MTCoff - MTCon]/MTCoff) allows detection of areas with high protein content and thus discrimination between thick collagen-I (MTR = 54%) and thin collagen-III fibers (MTR = 11%) as well as age classification of intraplaque hemorrhage (IPH). MTR of acute red cell rich IPH was 9%, of recent fibrin rich IPH 55% and of old IPH, rich in protein debris, was 69%. Surprisingly, lipid rich areas with relatively low protein content had an MTR of 46%. The authors conclude that MT CMR enhances plaque tissue contrast and identifies the protein-rich regions of carotid artery specimens. The additional information from MTR of IPH may provide important insight into the role of IPH on plaque stability, evolution, and the risk for future ischemic events.

### Assessment of the kidneys: magnetic resonance angiography, perfusion and diffusion

This review article addresses renal MRA and advanced renal MR techniques which is an interesting and important subject [103]. The current role of contrast agents and their safe use in patients with renal impairment is laid out and an insight beyond the current applications of renal MRA is provided. The clinical value and specific applications of renal MR are critically discussed. The author covers the subject well and offers a balanced appraisal of the techniques and their limitations as well as highlighting areas of future development. The article is well illustrated.

### Characterization of healing following atherosclerotic carotid plaque rupture in acutely symptomatic patients: an exploratory study using in vivo cardiovascular magnetic resonance

This paper describes the use of lumen curvature measurements obtained from MR images to characterize healing of ruptured plaque in individuals who have recently suffered from a transient ischemic attack [104]. Patients showing characteristics of plaque rupture on MR images were followed up after 3 and 12 months and the lumen curvature and roughness estimates were obtained. In individuals who did not have a follow up event, the plaque rupture showed signs of healing as demonstrated by reduction in maximum lumen curvature and improvement is surface roughness. The main limitations of the study include the small sample size especially in the group that had follow up events. The investigators therefore limited their analysis to only the group that did not have follow up events. Other limitations include the fact that the robustness and reproducibility of the approaches used have not been thoroughly validated.

### Carotid plaque regression following 6-month statin therapy assessed by 3 T cardiovascular magnetic resonance: comparison with ultrasound intima media thickness

This study compares CMR and B-mode ultrasound in monitoring changes in plaque burden over 6 months of statin therapy [105]. In 26 subjects, of whom 13 had initiation or increase in statin dosing, they found that plaque volume as measured by CMR significantly decreased by 5.8% whereas IMT remained unchanged. These findings support the concept of volumetric plaque acquisition as important step in the analysis of an asymmetric disease. The authors divided axial wall area into 6 regions and found that wall thickness differed between regions and the region with smallest wall thickness showed largest regression. The study is limited by the sample size and the variable statin dosing and regimen.

### Dilation of the ascending aorta in Turner syndrome - a prospective cardiovascular magnetic resonance study

This paper reports serial measurements of aortic dimensions in Turner syndrome patients and provides some insight into the degree of aortic growth and its associated risk factors [106]. The authors demonstrated a small but statistically significant growth rate (0.1 – 0.4 mm/yr) of the proximal aortic segments in the Turner population during 2.4 year mean follow up. The rate of growth at the sinus segment is demonstrated to be greater in those with BAV as opposed to trileaflet aortic valves. The authors conclude that a general aortopathy is present in TS with enlargement of the ascending aorta, which is accelerated in the presence of a bicuspid aortic valve.

### Magnetic resonance angiography: current status and future directions

This is an excellent review that covers all aspects of magnetic resonance angiography in some depth [107]. The overall background is good, the references adequate and the images are of uniformly excellent quality.

### Consistency of aortic distensibility and pulse wave velocity estimates with respect to the Bramwell-Hill theoretical model: a cardiovascular magnetic resonance study

The authors use a well-known model to derive theoretical values for aortic pulse wave velocity (PWV) from CMR-assessed data, and compared these with CMR-assessed values for PWV in the aortic arch [108]. CMR data was assessed in 46 healthy volunteers. Aortic stiffness is expressed in pulse wave velocity (PWV) of the aortic arch, and distensibility of the ascending aorta (determined by the local aortic area change and pulse pressure). Pulse pressure is estimated from a brachial pressure cuff-measurement and by carotid tonometry. The CMR-derived PWV in the aortic arch is compared with the theoretical local PWV (determined from distensibility assessment). The authors show good correlation between measured and theoretical PWV (r = 0.78). The paper is well written, the methods applied are appropriate and validated, although not new and the conclusions drawn from the results are appropriate.

### CMR Assessment of endothelial damage and angiogenesis in porcine coronary arteries using gadofosveset

This is a well-written study on the use of an albumin binding contrast agent for assessment of endothelial integrity in a pig model of coronary injury [109]. The authors validated their in-vivo and ex-vivo imaging findings of increased contrast uptake (~30 minutes post contrast agent injection) in the area of injury by Evan’s blue dye injection and subsequent histological analysis. Furthermore, staining for neovessels was performed and a correlation between neovessel density and ex-vivo contrast uptake was found. These are important findings as endothelial dysfunction is found both in early and late stages of atherosclerosis while angiogenesis is believed to be associated with plaque destabilization.

### Determination of edema in porcine coronary arteries by T2 weighted cardiovascular magnetic resonance

Although inflammation plays a key role in the progression of atherosclerotic plaques, it is the prediction of the next ‘culprit’ or ‘hot’ lesion with the potential to rupture giving rise to an acute coronary syndrome which is a major challenge in patients with coronary artery disease. Pedersen looked at edema in the proximal left anterior descending coronary artery in pigs following balloon injury using T2-weighted short tau inversion recovery (STIR) and conventional T2-weighted spin echo sequences [110]. STIR signal was much higher in the injured segments and detected edema with a sensitivity of 100% and a specificity of 71% using a threshold value for signal intensity of 7 SD above the average myocardial signal intensity. This correlated well with histological findings however, conventional T2-weighted images did not show significant changes in signal intensity post injury. This is a promising technique but poses a challenge in clinical practice due to the limitations of scanning small cross-sectional areas of the coronary artery.

### Multimodal cardiovascular magnetic resonance quantifies regional variation in vascular structure and function in patients with coronary artery disease: relationships with coronary disease severity

To date, most of the CMR studies looking at vascular parameters have focused on patients presenting with carotid disease [111,112]. Kylintireas studied 100 patients with known coronary artery disease, demonstrating atheromatous disease in various locations within the vascular tree [113]. In a multivariate analysis, carotid atheroma class, distal descending aorta atheroma burden, aortic distensibility and brachial artery reactivity correlated modestly with and were independent predictors of severity and extent of coronary artery disease, as expressed by the modified Gensini score. A comprehensive approach to CMR scanning is likely to extend our knowledge and understanding of the effects of atheroma and vascular disease throughout the cardiovascular system. This study provides an insight into which CMR parameters might be of further use with respect to the design of future clinical trials.

## Perfusion

Perfusion CMR has grown into a clinically important examination and is challenging established referral patterns for nuclear based techniques [114]. New steps in optimisation continue to be published [115,116] including accelerated acquisition, high field CMR, new stress protocols [117,118], assessment of new treatments [119,120], and improved analysis including quantification [121,122]. Progress in perfusion CMR in children and women in particular has occurred in a desire to lower radiation burden in these sensitive individuals. However there remains room for improvements in ease of analysis and quantification, artefact elimination, robustness and relation to outcomes.

### Diagnostic accuracy of adenosine stress cardiovascular magnetic resonance following acute ST-segment elevation myocardial infarction post primary angioplasty

Adenosine perfusion CMR is well established in chronic coronary disease, but its role in acute coronary disease is less well tested especially in the detection of non-culprit lesion ischemia. Wong et al studied 59 patients 3 days after acute ST-elevation myocardial infarction with 41% anterior infarctions and 100 non-culprit vessels [123]. The accuracy of perfusion CMR was 96%, with sensitivity of 99% and specificity of 67%. There was little diagnostic difference between visual and semi-quantitative analysis. The results suggest that perfusion CMR is diagnostically useful in the setting of acute infarction.

### Development of a universal dual-bolus injection scheme for the quantitative assessment of myocardial perfusion cardiovascular magnetic resonance

The dual bolus protocol for gadolinium injection for myocardial perfusion CMR enables accurate quantification but has disadvantages for complicated set-up. Ishida et al propose a simpler dual bolus regime which may have greater clinical utility [124]. The regime was tested on several MR scanners with different acquisition sequences, gadolinium compounds and doses, and was found to work well under these various conditions. The authors suggest that their regime could be used clinically obviating the need for complicated set up.

### Preliminary assessment of cardiac short term safety and efficacy of manganese chloride for cardiovascular magnetic resonance in humans

In contrast to Gadolinium chelates, manganese-containing agents are intracellular and manganese chloride is rapidly taken up into myocytes. Fernandes published preliminary safety and efficacy data regarding the use of manganese chloride in 17 healthy volunteers [125]. A significant reduction in T1 was observed in all subjects, sustained up to 30 minutes. The infusion was well tolerated with no major adverse events, however all patients reported a transient facial flush. This predictable, sustained reduction in T1 is described by the authors as a ‘memory effect’ that can be potentially explored to develop new imaging strategies.

## Acute coronary syndrome

CMR research in acute coronary syndromes has been stimulated by visualisation of pathological processes that are difficult [126] or impossible to image by other in-vivo techniques; This includes microvascular obstruction, myocardial edema, myocardial salvage, age of infarction [127] alternative diagnoses [128] and co-morbidities [129]. Improved techniques [130] and understanding of the events and their time course after acute coronary syndromes has potential to improve outcomes of primary percutaneous coronary intervention.

### Myocardial area at risk and salvage measured by T2-weighted cardiovascular magnetic resonance: Reproducibility and comparison of two T2-weighted protocols

The concept of myocardial area at risk and salvage in acute myocardial infarction is of major interest and key importance for clinical trials looking at interventions to reduce infarct size. Lønborg performed a comparison of two different T2-weighted CMR protocols to assess the myocardial area at risk and salvage index in 91 patients with acute ST-elevation myocardial infarction treated with primary percutaneous coronary intervention [131]. A second scan was performed at 3 months to look at final infarct size using a standard late gadolinium enhancement technique. The two STIR sequences each used a different slice thickness and echo time, detecting a statistically significant difference in the extent of myocardial oedema (and hence salvage index). Using a slice thickness of 15 mm picked up a larger area at risk than the sequence with a slice thickness of 8 mm (p < 0.01). Whilst the authors were not able to provide a specific recommendation for one sequence over the other, this study underlines the importance of understanding the specific protocol used for assessment of area at risk and its potential impact on the results of clinical trials.

## Chronic ischemic heart disease

The use of late gadolinium enhancement (LGE) has transformed the investigation and clinical practice of chronic coronary disease, and recently yielded new insights into infarction, viability, medical treatment [132] and revascularisation [133]. Work is still progressing on how best to quantify LGE in relation to outcome, and the relative merits versus dobutamine stress CMR [134]. The JCMR papers presented examine important aspects of this field.

### The role of dobutamine stress cardiovascular magnetic resonance in the clinical management of patients with suspected and known coronary artery disease

Dobutamine stress CMR is effective in the identification of myocardial ischemia, but often in the research setting. Gebker et al report a large study of 1532 patients to determine the value of the technique in the routine clinical setting [135]. Patients with positive stress CMR were recommended to undergo coronary angiography and those with negative stress CMR received optimal medical therapy. Of 609 positive patients, 478 (78%) had coronary angiography within 90 days and of these 409 (89%) had significant coronary stenosis. of 923 negative patients, only 8 patients (0.96%) had a cardiac event during a mean follow-up period of 2.1 years. In 131 positive patients who did not undergo coronary angiography, 20 (15%) patients had a cardiac event. The authors conclude that dobutamine stress CMR has substantial clinical utility in the general clinic setting.

### Prevalence of scarred and dysfunctional myocardium in patients with heart failure of ischaemic origin: A cardiovascular magnetic resonance study

Ischaemic heart disease is a common cause of left ventricular systolic dysfunction which can lead to chronic heart failure. The role of revascularisation for potentially hibernating areas of myocardium remains unclear but previous studies of heart failure patients with evidence of significant viability have been neutral in terms of hard endpoints and have not included late gadolinium CMR for assessment of viability [136-138]. It is in this context that Bourantas scanned a cohort of 193 patients with evidence of ischaemic heart disease and LV ejection fraction < 50% [139]. Myocardial contractility and transmural extent of scar were assessed using a 17-segment model. Although approximately half of all myocardial segments showed contractile dysfunction, only one third of these had > 50% of the wall thickness affected by scar, suggesting that most could improve in response to an appropriate intervention. Further research is required to determine whether the extent of myocardial scar as measured by late gadolinium CMR can be used to predict the likely extent of recovery in ventricular function with pharmacological interventions and revascularisation.

### Value of scar imaging and inotropic reserve combination for the prediction of segmental and global left ventricular functional recovery after revascularisation

Previous authors have noted the utility of adding a low-dose dobutamine stress protocol to conventional scar imaging in order to improve the potential predictive value of CMR for assessment of hibernation prior to revascularisation [140]. Glaveckaite studied the combination of low dose dobutamine (to assess contractile reserve) together with transmurality of late gadolinium enhancement and the thickness of the residual viable ‘rim’ of myocardium in 46 patients with coronary artery disease before and after revascularisation [141]. Baseline LV ejection fraction was 35 ± 8%. Receiver operator curve analysis showed that a combined model of low dose dobutamine and infarct transmurality gave the strongest sensitivity and specificity for prediction of improvement in LV ejection fraction following revascularisation (area under the curve 0.84, p < 0.001). This paper adds to the growing weight of literature regarding CMR assessment of viability and likelihood of functional myocardial recovery after successful revascularisation.

### Effect of ischemic preconditioning in skeletal muscle measured by functional magnetic resonance imaging

Ischaemic preconditioning (both in the myocardium and in remote organs) has been shown to mitigate ischemia-perfusion injury, thereby reducing infarct size following coronary artery occlusion [142,143]. Using a model of leg ischemia in healthy subjects at 3 T, Andreas compared 31P MR spectroscopy and blood oxygen level dependence (BOLD) with isometric muscle strength [144]. Ischemic preconditioning 4 hours prior to a period of ischemia significantly increased the maximal phosphocreatine (PCr) signal (p < 0.05) and lowered the peak BOLD signal during hyperaemic reperfusion (p < 0.05). This suggests a positive influence on muscle metabolism during reperfusion with an increase in PCr production and higher oxygen consumption. Mimicking arterial stenosis with low-flow reperfusion prevented the recovery of PCr and was associated with a decrease in muscular strength, thus highlighting the importance of full and rapid reperfusion. This promising work indicates that functional MR can provide an objective assessment of changes in muscle metabolism following reperfusion and therapeutic interventions in vivo. If this can be reliably extrapolated to the heart, it could be a valuable tool for assessment of myocardial ischemia-reperfusion models in patients.

### Prevalence and prognosis of myocardial scar in patients with known or suspected coronary artery disease and normal wall motion

In contrast to CMR which has a high sensitivity and specificity for detecting myocardial infarction using LGE imaging, other non-invasive imaging techniques can miss subtle subendocardial infarcts [145]. Krittayaphong takes this one stage further, looking at 1148 patients with suspected or known coronary artery disease who had normal left ventricular wall motion [146]. Multivariate analysis was used to look at the association of a panel of clinical factors, medications prescribed, ECG detection of myocardial infarction and CMR parameters (presence of late enhancement, LV volumes and mass) with outcomes. LGEwas detected in 104 patients (9.1%) and over an average follow-up of 955 ± 542 days, the presence of late enhancement was the strongest predictor of outcomes in terms of hard endpoints (cardiac death and nonfatal myocardial infarction, p = 0.004) and major adverse cardiac events (p < 0.001). It cannot therefore be assumed that the absence of a left ventricular wall motion abnormality excludes underlying structural heart disease, thereby indicating a good prognosis.

## Technical advances and new techniques

The editors of JCMR continue to support publication of new CMR techniques involving new sequences [147-149], applications [150-153], animal models [154-156] and analysis techniques [157]. Recent review articles have proven very popular [158-160] and we have continued to commission such articles in new fields. These new techniques described in this section are of interest especially to the CMR physics community for translation into robust new human tools.

### Quantitative comparison of myocardial fiber structure between mice, rabbit, and sheep using diffusion tensor cardiovascular magnetic resonance

The fundamental understanding of cardiac structure and function requires structural models of the heart, but little is known about interspecies structure variation. Using diffusion tensor imaging (DTI), which was recently reviewed in JCMR [161], Healy scanned mouse, rabbit and sheep hearts after fixation and quantitatively assessed fiber orientation and the transmural range and linearity of fiber helix angles [162]. The authors showed significant differences between species and argue that caution must be exercised in extrapolating results between animals.

### Comprehensive Cardiovascular magnetic resonance of myocardial mechanics in mice using three-dimensional cine DENSE

This paper describes the development and implementation of a 3D cine DENSE pulse sequence on a 7 T small-bore scanner for the study of micromechanics in mice [163]. This highly sophisticated MR technique used three-point phase cycling for artifact suppression and a stack-of-spirals k-space trajectory for efficient data acquisition. Using these methods, multiphase normal and shear strains were measured, as were myocardial twist and torsion and the resulting 25-min acquisition time represents a huge improvement over the currently used 2D methods.

### Non-triggered quantification of central and peripheral pulse-wave velocity

This paper represents the latest development in methods for measurement of arterial pulse wave velocity (PWV) [164]. The MR sequence is “real-time” and therefore doesn’t require cardiac triggering so that it can be used to monitor PWV changes over several cycles. The method which "simultaneously" excites and collects a series of velocity-encoded projections at two arterial segments to estimate the wave-front velocity was used to study PWV between the aortic arch and iliofemoral arteries in normal subjects. This appears to be the first to demonstrate variations in PWV between cardiac cycles.

### Simultaneous mapping of temporally-resolved blood flow velocity and oxygenation in femoral artery and vein during reactive hyperemia

The outcome of the research described in this manuscript was an integrated study of flow and oxygen saturation for improved assessment of vascular disease [165]. The authors describe a method to assess the hyperemic response in the femoral artery by measuring changes in both flow and oxygen concentration by use of a combination of velocity-encoded projections and multi-echo susceptibility weighted imaging. It is shown that multiple parameters may be quantified enabling more detailed assessment of peripheral vascular reactivity in a single cuff paradigm rather than in separate procedures as generally required, thus improving study efficiency and patient comfort.

### Acceleration of tissue phase mapping with sensitivity encoding at 3 T

Despite a number of advantages a particular problem with phase contrast velocity mapping of the myocardium is the long acquisition times. This manuscript describes a study to assess the impact of using frame-by-frame SENSE to accelerate the acquisition of such maps and therefore make them more clinically applicable [166]. The work was done on a 3 T scanner with a 32 channel receiver coil and the results show that even with an acceleration factor of 4 there is minimal impact of the measured myocardial velocities throughout the cardiac cycle.

### Regional contrast agent quantification in a mouse model of myocardial infarction using 3D cardiac T1 mapping

This manuscript describes the application of a recently developed 3D T1 mapping technique in the mouse to study myocardial infarction and to measure differences in myocardial T1 before and after injection of a liposomal contrast agent [167]. This was then used to assess the concentration of accumulated contrast agent which was compared and correlated to ex vivo concentrations determined by ICP-MS. The manuscript represents a further progression in the development of techniques available to those wishing to study heart in such models.

### Quantification and visualization of cardiovascular 4D velocity mapping accelerated with parallel imaging or k-t BLAST: head to head comparison and validation at 1.5 T and 3 T

This manuscript is a validation of quantitative in vivo cardiac 4D flow measurements where the acquisition has been accelerated with parallel imaging and k-t BLAST at 1.5 T and 3 T [168]. These techniques are conceptually appealing because they allow flow measurements to be made in any plane after the patient has left the magnet. Potentially, if the scans can be accelerated, this could be important for evaluating complicated congenital heart disease. The results of this study show that although the accuracy of 4D flow is comparably good at 1.5 and 3 T the acceleration methods both resulted in an underestimation of velocity. For flow visualisation, however, all methods produced similar quality.

### Accelerating global left-ventricular function assessment in mice using reduced slice acquisition and three-dimensional guide-point modelling

There is a general problem of long acquisition and analysis times for measurement of cardiac function in mouse models. In this study guide-point modelling was used with reduced slice numbers reducing acquisition times for the determination of left ventricular function parameters in the infarcted mouse [169]. The study tested the hypothesis that a reduced the number of slices could be acquired and for accurate determination of left ventricular function using guide-point modelling. The results confirmed the method allowed accurate analysis of function in mice with relatively large infarcts using a reduced slice protocol and that a further reduction was possible in mice with a normal left-ventricular topology.

### Rapid assessment of myocardial infarct size in rodents using multi-slice inversion recovery late gadolinium enhancement CMR at 9.4 T

This manuscript describes another development of improved cardiac imaging in the small animal rodent model [170]. In this case the authors have developed a multi-slice inversion recovery technique produces high quality images with excellent infarct definition in a short acquisition time. The technique was applied to two preclinical scenarios of an acute reperfused model of MI in rats and also mice 2 days following non-reperfused MI. The study showed how the technique could be adapted and optimised for the different models for and in rats the method showed close agreement for infarct sizing when compared to histological staining.

### First-pass perfusion CMR two days after infarction predicts severity of functional impairment six weeks later in the rat heart

The authors present a study on first-pass perfusion in a small animal model, stating that perfusion and infarct size may predict severity of impairment [171]. Perfusion imaging in the rapidly beating rodent heart is challenging and this study tackles some of the issues. A ‘novel’ and ‘simple’ perfusion method is introduced by acquiring a 64 × 64 pixels image and zero filling k-space to a 256 × 256. The study showed that the perfusion delay was larger in rat hearts that went on to develop greater functional impairment, demonstrating that first-pass CMR can be used as an early indicator of infarct severity.

### Myocardial tagging by Cardiovascular Magnetic Resonance: evolution of techniques--pulse sequences, analysis algorithms, and applications

This excellent well referenced review describes in an extremely thorough way all the tagging sequences, their advances and how the different approaches interrelate primarily from a technical perspective but also with an interesting historical context [172]. The figures explaining the techniques are also of high quality and helpful for understanding. In a similar way the work also details and compares the various methods of tagging analysis. This is very much a technical review and clinical applications and results await future review.

### Acceleration of tissue phase mapping by k-t BLAST: a detailed analysis of the influence of k-t-BLAST for the quantification of myocardial motion at 3 T

This manuscript describes a systematic study of the impact of k-t BLAST on the measurement on myocardial function by tissue phase mapping [173]. Myocardial velocity measurements were compared with every acceleration factor between 2 and 7. Interestingly the results indicate that even with an acceleration of as little as two there is a marginal, but statistically significant deterioration of the velocity peaks and this is increased with further acceleration. The temporal behaviour of the motion, however, was well maintained up to an acceleration factor of six.

## Varia

There is a well recognised source of referrals which is simply put as unusual pathology, or cases where other imaging has failed to yield a definitive diagnosis. These include for example pericardial disease, tumours [174], and inflammatory diseases. We therefore include this section on papers and also include official reports, guidelines and President's Pages [175,176] which are not readily categorized.

### Magnetic resonance imaging in patients with cardiac pacemakers: era of "MR Conditional" designs

Advances in cardiac device technology have led to the first generation of MR conditional devices. This timely paper reviews the current state of the art, the likely future of new devices and the problems in development [177].

### Impact of an abdominal belt on breathing patterns and scan efficiency in whole-heart coronary magnetic resonance angiography: comparison between the UK and Japan

Long acquisition times and complex breathing patterns limit the clinical applicability of whole heart 3D coronary MR angiography. Ishida et al designed and tested a tight abdominal belt in 15 Japanese and 15 English patients, to determine whether the belt could improve diaphragmatic position [178]. The authors showed that scan efficiency was significantly improved in both cohorts suggesting possible clinical utility.

### Cardiovascular magnetic resonance activity in the United Kingdom: a survey on behalf of the British society of cardiovascular magnetic resonance

Antony et al conducted a survey of 60 National Health Service providers of CMR in the UK and obtained replies from 88% [179]. There were equal numbers of cardiologists and radiologists in leadership positions. Scan volumes had increased by 44% over the previous 2 years. The commonest indication for CMR was heart failure and cardiomyopathy (39%) followed by coronary artery disease and congenital disease. Formal training programs existed in about half of centres. This survey shows that CMR is increasing rapidly in the UK and is well disseminated, although there are some very large volume specialist centres.

### Gender differences in response to cold pressor test assessed with velocity-encoded cardiovascular magnetic resonance of the coronary sinus

Moro et al measured coronary sinus flow during cold pressor stress to determine whether gender differences could be found reflecting coronary endothelial function [180]. The authors studied 12 normal men and 12 women at 3 T and showed that despite similar baseline coronary sinus flow, that women increased flow significantly more than men with stress. This test might prove useful for assessing coronary endothelial function.

## Abbreviations

ACEI: Angiotensin converting enzyme inhibitor; ARB: Angiogtensin receptor blocker; AVA: Aortic valve area; BOLD: Blood oxygen level dependent; CMR: Cardiovascular magnetic resonance; CRT: Cardiac resynchronisation therapy; CT x-ray: Computed tomography; DCM: Dilated cardiomyopathy; DMD: Duchenne muscular dystrophy; DTI: Diffusion tensor imaging; EF: Ejection fraction; ECV: Extracellular cardiac volume; HCM: Hypertrophic cardiomyopathy; IP: Intraplaque hemorrhage; JCMR: Journal of cardiovascular magnetic resonance; LGE: Late gadolinium enhancement; LGMD: Limb-girdle muscular dystrophy; LV: Left ventricle; LVOT: Left ventricular outflow tract; MTC: Magnetisation transfer contrast; MRA: Magnetic resonance angiography; PCr: Phosphcreatine; PWV: Pulse wave velocity; RV: Right ventricle; SSFP: Steady state free precesson; STIR: Short tau inversion recovery; TTE: Transthoracic echo; TAVI: Transcatheter aortic valve implantation; VF: Ventricular fibrillation; 3D: 3 dimensional; 4D: 4 dimensional.

## Competing interests

The authors declare that they have no competing interests.

## Authors’ contributions

All authors contributed to the writing of this review article. All authors read and approved the final manuscript
